# An Optical AC Voltage Sensor Based on the Transverse Pockels Effect

**DOI:** 10.3390/s110706593

**Published:** 2011-06-27

**Authors:** Feng Pan, Xia Xiao, Yan Xu, Shiyan Ren

**Affiliations:** State Key Laboratory of Advanced Electromagnetic Engineering and Technology, College of Electrical and Electronic Engineering, Huazhong University of Science and Technology, 1037 Luoyu Road, 430074 Wuhan, China; E-Mails: xiaoxiahust@163.com (X.X.); xuyan919@mail.hust.edu.cn (Y.X.); shiyan@public.wh.hb.cn (S.R.)

**Keywords:** optical voltage sensor, electro-optical crystal, Bi_4_Ge_3_O_12_, transverse Pockels effect

## Abstract

This paper introduces an optical AC voltage sensor based on the transverse Pockels effect. The sensor utilizes a bulk Bi_4_Ge_3_O_12_ (BGO) crystal as the sensing element. The measurement principle has been described and prototype of the sensor has been constructed and evaluated. Good linearity and accuracy performance was obtained for AC voltage measurement. The proposed sensor can be thus applied to high AC voltage measurements in the electric power industry.

## 1. Introduction

Optical voltage sensors (OVS) for AC voltage measurement have been investigated for many years. Their applications in the electric power industry have also been addressed [1–4]. In contrast to conventional voltage sensor technology (e.g., inductive voltage transformers or capacitive voltage transformers), OVS has inherent and advantageous features, such as wider bandwidth, larger dynamic range and lighter weight. Light transmission to and from the region of voltage measurement is done by optical fibers which bring inherent immunity to electromagnetic interference and compatibility with low voltage analog and digital equipment used for metering and relaying applications.

The Pockels effect (or linear electro-optic effect) is an electric field-induced linear birefringence or anisotropic change in the refractive index of the material. The Pockels effect can be used in two modes. When the applied electric field is normal to the direction of propagation of the incident light, the transverse Pockels effect is said to occur. When the applied field and the propagation direction are parallel, the longitudinal Pockels effect is said to take place [5,6].

Most optical voltage sensors are based on an electro-optic (EO) crystal and longitudinal Pockels effect. The problem for this case is the sensitivity of EO crystal, which is usually too high in comparison with the measured voltage. The conventional solution is to use capacitive dividers to obtain a small part of the total voltage on the optical voltage sensor [7]. However, the method limits the performance of the optical measurement technology due to the high cost and the stability problem of the capacitive dividers. Another method is to use the multi-segmented sensor which consists of crystal slices and spacers of dielectric material [8]. The half wave voltage of the multi-segmented sensor is far larger than a single EO crystal in longitudinal modulation.

In this paper, an optical voltage sensor based on the transverse Pockels effect is introduced. This sensor does not need any capacitive voltage dividers and can directly measures the electrical field strength between the high potential electrode and ground electrode. The principle of the OVS has been introduced and a prototype of the sensor has been constructed and tested.

## 2. OVS Design

### 2.1. Principle

**Figure 1.**
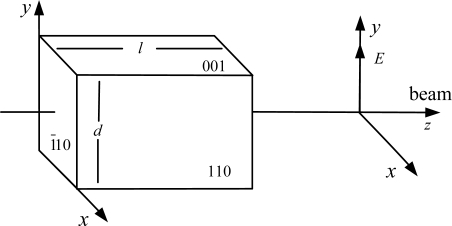
Orientation of the BGO crystal used in the OVS.

We have selected Bi_4_Ge_3_O_12_ as the electro-optic crystal. It is a cubic crystal of point group symmetry 4̄3*m* and does not exhibit natural linear birefringence [9]. The orientation of BGO crystal used in the OVS is shown in Figure 1. When an electric field is applied on the BGO crystal along the [001] direction, the principle indexes of refraction are given by:

(1) $$n_{x}=n_{0}-\frac{1}{2}n_{0}^{3}\gamma_{41}E$$

(2) $$n_{y}=n_{0}$$

(3) $$n_{z}=n_{0}+\frac{1}{2}n_{0}^{3}\gamma_{41}E$$

where *n_o_* is the refraction index of the ordinary ray, *γ*_41_ is the electro-optic coefficient and *E* is the value of the applied electric field strength.

When a linearly polarized light enters into the BGO crystal along the *z*-axis, it can be treated as two orthogonally linearly polarized lights along the *x*-axis and *y*-axis, separately. As the two lights propagate within the crystal, they will experience different phase delays, which can be related to the optical path length difference. The phase delay is given by:

(4) $$\delta_{E}=\frac{2\pi }{\lambda }(n_{y}-n_{x})l=\frac{\pi }{\lambda }n_{0}^{3}\gamma_{41}lE$$

where *λ* is the light wavelength and *l* is the length of the crystal along the light propagation direction.

If the electric field is obtained by applying a voltage *V* on the two faces of the BGO crystal, the induced phase retardation is:

(5) $$\delta_{V}=\frac{\pi }{\lambda }n_{0}^{3}\gamma_{41}l\cdot \frac{V}{d}=\frac{\pi V}{V_{\pi }}$$

where *d* is the thickness of the crystal along the electric field direction and the parameter *V_π_*, known as the half-wave voltage, is the applied voltage at which the phase delay is *π*. The value of *V_π_* is given by:

(6) $$V_{\pi }=\frac{\lambda d}{n_{0}^{3}\lambda_{41}l}$$

### 2.2. OVS Setup

**Figure 2.**
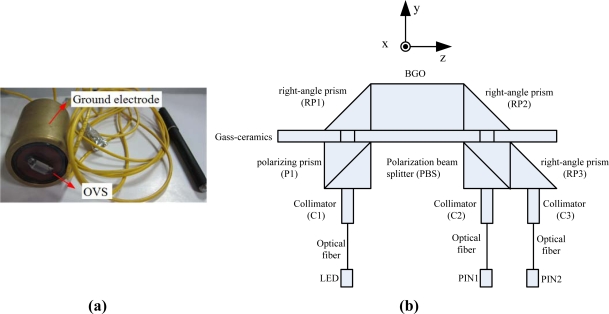
**(a)** Photo of the OVS. **(b)** Setup of the OVS.

The photo and setup of the OVS are shown in Figure 2. The BGO crystal is placed on the upper surface of a glass-ceramics and the [001] direction is parallel to the *y*-axis.

The light beam coming from a light emitting diode (LED) is sent to the sensor through the optical fiber. The light beam enters into the fist collimator (C1), then passes the polarizing prism (P1) and travels through the glass-ceramics. The linearly polarized light is internally reflected in the first right-angle prism (RP1). When the reflected polarized light enters the BGO crystal, a phase difference that is lineally proportional to the external electric field strength is introduced. At the second right-angle prism (RP2), a totally internal reflection takes place directing the beam of light through the polarization beam splitter (PBS) and right-angle prism (RP3). The PBS splits the merging light intensity into two components. Finally, after passing through collimators (C2 and C3) and optical fibers, the two paths of sensing signals are detected by the PIN photodiodes (PIN1 and PIN2).

To analyze the transmitted intensity through the whole sensor, we use Johns calculus [10]. Considering P1 passes only the wave component that is linearly polarized at angle *α* to the *x* axis, the Jones matrix for P1 is:

(7) $$J_{P1}=\frac{\sqrt{2}}{2}\left[\begin{array}{l} \text{cos}\;\alpha \\ \text{sin}\;\alpha \end{array}\right]$$

The RP1 and RP2 (made of BAK4 and PSK3, respectively) can produce a phase retardation angle of 90 degrees [11]. Assuming the phase retardation angles are *ϕ*_1_ and *ϕ*_2_ for RP1 and RP2 respectively, the Jones matrixes are given by:

(8) $$J_{RP1}=\left[\begin{array}{cc} 1 & 0\\ 0 & e^{-i\varphi_{1}} \end{array}\right]$$

(9) $$J_{RP2}=\left[\begin{array}{cc} 1 & 0\\ 0 & e^{-i\varphi_{2}} \end{array}\right]$$

Considering the BGO crystal introduces phase delay *δ_V_*, the Johns matrix for BGO can be written as:

(10) $$J_{B}=\left[\begin{array}{cc} 1 & 0\\ 0 & e^{-i\delta_{V}} \end{array}\right]$$

We consider the PBS passes only the wave component that is linearly polarized at angle *β* to the *x* axis. The Jones matrix for such a device is given by:

(11) $$J_{P2}=\left[\begin{array}{cc} {\text{cos}}^{2}\;\beta & \text{sin}\;\beta \;\text{cos}\;\beta \\ \text{sin}\;\beta \;\text{cos}\;\beta & {\text{sin}}^{2}\;\beta \end{array}\right]$$

It can easily be shown that the transmitted intensity through the whole sensor is given by:

(12) $$\vec{E_{o}}=\left[\begin{array}{c} E_{op}\\ E_{os} \end{array}\right]=J_{P2}\cdot J_{RP2}\cdot J_{B}\cdot J_{RP1}\cdot J_{P1}\cdot \vec{E_{i}}$$

When *ϕ*_1_ + *ϕ*_2_ = 90°, *α* = 45°, *β* = ±45°, the transmitted intensity is:

(13) $$\vec{E_{o}}=\frac{\sqrt{2}}{4}\left[\begin{array}{l} 1\pm e^{-i(\delta_{V}+90{}^{\circ})}\\ \pm 1+e^{-i(\delta_{V}+90{}^{\circ})} \end{array}\right]\vec{E_{i}}$$

The emergent light intensity is:

(14) $$\begin{array}{l} I_{o}=\vec{E_{o}^{*}}\cdot \vec{E_{o}^{*}}\\ =\frac{1}{2}I_{i}[1\pm \text{cos}(90{}^{\circ}+\delta_{V})]\\ =\frac{1}{2}I_{i}(1\mp \text{sin}\;\delta_{V}) \end{array}$$

where $I_{i}=E_{i}^{2}$ is the incident light intensity.

For the small value of *δ_V_*, we can take the approximation of sin *δ_V_* ≈ *δ_V_*. Thus the emergent light intensity is:

(15) $$I_{o}=\frac{1}{2}I_{i}(1\mp \text{sin}\;\delta_{V})\approx \frac{1}{2}I_{i}(1\mp \delta_{V})$$

In order to ensure the error of approximate calculation in the Equation 15 is less than 0.1%, the range of voltage applied on the BGO can be estimated by the following equation:

(16) $$\left|\frac{\delta_{V}-\text{sin}\;\delta_{V}}{\delta_{V}}\right|\approx \frac{\delta_{V}^{2}}{6}<0.001$$

Thus the voltage applied on the BGO should meet the following condition:

(17) $$V<0.0246\cdot V_{\pi }$$

In the proposed OVS, the sensed signal must be calculated from the measured light intensity that comes out of the sensor. Considering the crystals of class 4̄3*m* exhibit unwanted linear birefringence due to strain, stress or precipitates [12], the intensities of the emergent lights are of the form:

(18) $$I_{o1}=\frac{1}{2}I_{i}[1-(\delta_{V}+\delta_{U})]$$

(19) $$I_{o2}=\frac{1}{2}I_{i}[1+(\delta_{V}+\delta_{U})]$$

where *δ_V_* is the phase retardation introduced by the electric field, *δ_U_* is the phase retardation due to the unwanted birefringence.

### 2.3. The Signal Processing Circuit

**Figure 3.**
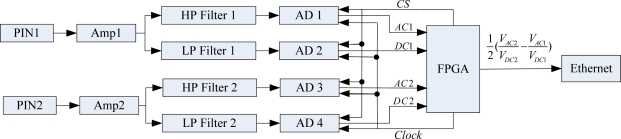
Block diagram of the signal processing circuit.

The schematic diagram of the signal processing circuit is shown in Figure 3. The photocurrent signals were converted into voltages and amplified by the analog amplifiers. High pass (HP) filters and low pass (LP) filters were utilized to acquire the AC and DC components of the sensing signals from two light paths. The AC and DC components are simultaneously sampled by analog to digital converters (AD1, AD2, AD3 and AD4). The AD converters operate at 4 kHz with 16 bits of resolution.

By dividing the AC component by the DC one in the Field-programmable Gate Array (FPGA), we obtain the ratios as follows:

(20) $$\frac{V_{AC1}}{V_{DC1}}=k_{1}\frac{-\delta_{V}}{1-\delta_{U}}\approx -k_{1}\delta_{V}(1+\delta_{U})$$

(21) $$\frac{V_{AC2}}{V_{DC2}}=k_{2}\frac{\delta_{V}}{1+\delta_{U}}\approx -k_{2}\delta_{V}(1-\delta_{U})$$

where *k*_1_ and *k*_2_ are the constants associated with the photodiodes responsibilities and electronic mismatches.

When *k*_1_ ≈ *k*_2_, we obtain the output signal of the sensor system as follows:

(22) $$V_{ovs}=\frac{1}{2}(\frac{V_{AC2}}{V_{DC2}}-\frac{V_{AC1}}{V_{DC1}})=k_{1}\delta_{V}=k_{2}\delta_{V}$$

## 3. Experiments and Results

### 3.1. Experiment Setup

The major optical properties of the BGO crystal at *λ* = 850 nm are *n*_0_ = 2.07 and *γ*_41_ = 1.03 × 10^−12^ m/V. The size of the BGO crystal is 5 × 5 × 10 mm^3^. According to the Equation 6, the half-wave voltage is *V_π_* = 46.52 kV. The voltage applied on the BGO crystal should be less than 1,144 V according to Equation 17.

**Figure 4.**
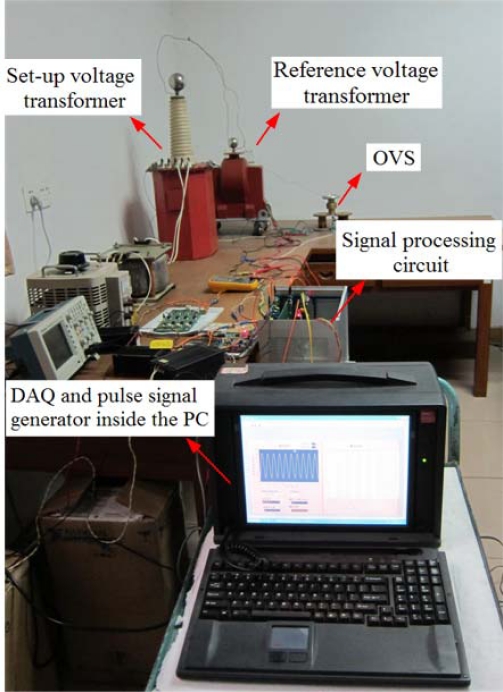
Experimental setup of the OVS.

The experimental setup is shown in Figure 4. The AC voltage is generated by a set-up voltage transformer which is connected to the high voltage potential electrode. The other electrode was connected to the ground potential. Between the two electrodes an electric field is created. By changing the shapes of the electrodes and the distance between the two electrodes, it is possible to make the value of the measured voltage adjustable and make sure that the applied voltage on the BGO crystal is less than 1,144 V. The parameters of the electrodes are obtained in advance by the finite element method (FEM).

For measuring and metering purpose, the ratio error and phase error at the voltage between 80% and 120% of the rated voltage should be considered [13]. We set the rated voltage of the OVS prototype to be 10 kV (the root mean square value, rms). Then the measurement range of the OVS prototype is from 8 kV to 12 kV (rms).

The OVS is sensitive to the electric field and the digital output of the OVS system is directly transmitted into the personal computer (PC). The measured high voltage is transformed into a low one by means of a reference voltage transformer. Then the low voltage is sampled by a data acquisition board (DAQ) and the digital data is transmitted into the PC.

A pulse signal generator accommodates an external sampling signal for the FPGA and DAQ, ensuring the data from the OVS system and the reference voltage transformer time-coherent. The ratio error and phase error, namely the deviation between the OVS system measurement and the reference voltage transformer measurement, are given by the DFT algorithm in the Labview programming environment. The ratio error expressed in percent is given by the formula:

(23) $$ɛ(\%)=\frac{k_{ovs}|V_{ovs}|-k_{ref}|V_{ref}|}{k_{ref}|V_{ref}|}\cdot 100$$

where |*V_ovs_*| is the amplitude value of the OVS system output, |*V_ref_*| is the amplitude value of the reference voltage transformer output, *k_ovs_* is the transformation ratio of OVS system, *k_ref_* is the transformation ratio of reference voltage transformer.

The phase error is expressed by:

(24) $$\varphi_{e}=\varphi_{ovs}-\varphi_{ref}$$

where *ϕ_ovs_* is the phase of *V_ovs_*, *ϕ_ref_* is the phase of *V_ref_*.

### 3.2. Results

**Figure 5.**
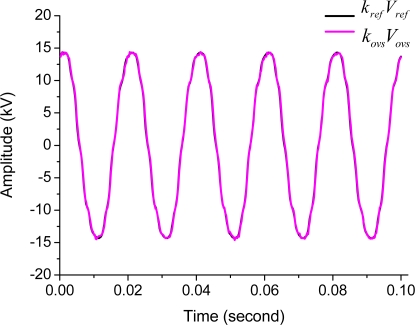
Waveforms of the reference voltage transformer measurement and the OVS system measurement.

The waveforms of the reference voltage transformer measurement and the OVS system measurement when the measured voltage is 10 kV (rms) are shown in the Figure 5. The results show that the waveforms are in good agreement between the reference voltage transformer and the OVS system.

**Figure 6.**
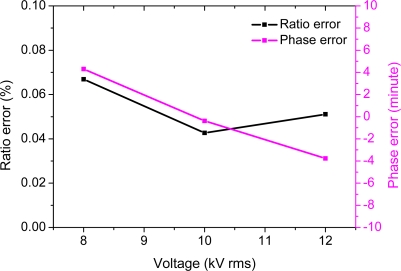
Ratio error and phase error *versus* applied voltage from 8 kV to 12 kV (rms).

The ratio error and phase error of the OVS *versus* voltage from 8 kV to 12 kV (rms) are shown in Figure 6. The results indicate that the ratio error is within ±0.1% and the phase error is within ±8 min. A good linear relationship between the output of the reference voltage transformer and the output of the tested OVS system is obtained.

**Figure 7.**
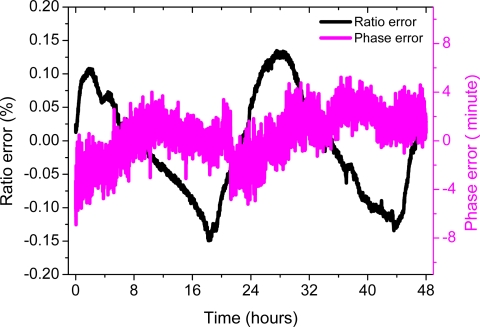
Ratio error and phase error *versus* time at a rated voltage of 10 kV (rms).

Figure 7 shows the errors of the sensor at a rated voltage of 10 kV (rms) in 48 h period. The ratio error is within ±0.15% and the phase error is within ±8 min. The results indicate that the stability of the OVS system is good.

## 4. Conclusions

An optical voltage sensor based on the transverse Pockels effect is introduced in this paper. The sensor directly measures the electrical field strength between a high potential electrode and a ground electrode without capacitive dividers. The sensor has good linearity in the voltage range from 8 kV to 12 kV (rms). The output of the sensor system is stable at the rated voltage in the laboratory environment. By changing the shapes of the electrodes and the distance between the two electrodes, it is possible to make the measurement range of the OVS adjustable. Therefore the proposed sensor is sufficiently satisfactory for measuring and metering application in different voltage levels.

## References

[1] Mitsui T, Hosoe K, Usami H, Miyamoto S (1987). Development of Fiberoptic Voltage Sensors and Magnetic-Field Sensors. IEEE Trans. Power Deliv.

[2] Laurensse IJ, Koreman CGA, Rutgers WR, Van der Wey AH (1989). Applications for Optical Current and Voltage Sensors. Sens. Actuat.

[3] Sawa T, Kurosawa K, Kaminishi T, Yokota T (1990). Development of Optical Instrument Transformers. IEEE Trans. Power Deliv.

[4] Bohnert K, Gabus P, Kostovic J, Brandle H (2005). Optical fiber sensors for the electric power industry. Opt. Laser. Eng.

[5] López-Higuera JM (2002). Handbook of Optical Fibre Sensing Technology.

[6] Gupta SC (2005). Optoelectronic Devices and Systems.

[7] (1997). Optical H.V. Sensors 123 to 765 kV, Balteau Series CTO-VTO-CCO.

[8] Santos JC, Taplamacioglu MC, Hidaka K (2000). Pockels High-Voltage Measurement System. IEEE Trans. Power Deliv.

[9] Williams PA, Rose AH, Lee KS, Conrad DC, Day GW, Hale PD (1996). Optical, Thermo-Optic, Electro-Optic, and Photoelastic Properties of Bismuth Germanate (Bi_4_Ge_3_O_12_). Appl. Opt.

[10] Goodman JW (2005). Introduction to Fourier Optics.

[11] Fabricius H (1991). Achromatic Prism Retarder for Use in Polarimetric Sensors. Appl. Opt.

[12] Lee KS (1989). New Compensation Method for Bulk Optical Sensors with Multiple Birefringences. Appl. Opt.

[13] (1999). Instrument Transformer—Electronic Voltage Transformers.

